# Supplementary material for: Reliable machine learning models in genomic medicine using conformal prediction
Source: Front Bioinform. 2025 Feb 24;5:1507448. doi: 10.3389/fbinf.2025.1507448 (PMC11891349; doi:10.3389/fbinf.2025.1507448)
Supplement: Supplementary file 1 [file DataSheet1.pdf]

Supplementary file
Conformal prediction framework

1 Conformal prediction frameworks

Conformal Prediction (CP) is defined as a mathematical framework that can be used with any Machine Learning (ML) model to produce reliable predictions with high probability and user-defined error rates (Shafer & Vovk, 2008; Vovk et al., 2005). Given a set of training data D, with n instances $\{(x_i, y_i), ..., (x_n, y_n)\}$, where x_i is a feature vector and y_i is the true label of the i-th sample, with K labels in $Y = [1, K]$, the objective is to predict the label $y_{n+1} \in Y$ for a new sample with feature vector x_{n+1}. In classification problems, we test all K classes of a new instance and measure the probability of a prediction to be the correct one for each class. To do so, we calculate the *non-conformity* score α_i, which is based on the underlying ML algorithm and indicates how strange an instance is compared with other instances of the same class. A simple example of a *non-conformity* score is the *1-predicted probability of the true class*, otherwise called inverse probability. Based on the hypothesis that the instances are independent and identically distributed random variables (i.i.d.), for a new instance x_{n+1} we compute the *non-conformity* score α^y_{n+1} for each possible class. Finally, for each possible label we calculate the *p-value* as:

$$p - value_y = \frac{\sum \left\{\alpha_i \geq \alpha^y_{n+1}\right\} + 1}{n+1}, \forall i \in \{1, .., n\} \tag{1}$$

p-value is used to evaluate the *non-conformity* score of the new instance α_{n+1} against all other *non-conformity* scores. False predictions result in a higher α_{n+1} than the rest *non-conformity* scores of the training set. In this case, we get a low *p-value*, while in cases of correct prediction, we expect a higher *p-value*. So, for a dataset that satisfies the i.i.d. assumption, every *p-value* in Eq. 1 has the following property validity guarantee:

$$P(p - value_y \leq \epsilon) \leq \epsilon \tag{2}$$

where, ϵ is the user-defined significance level (or target probability error). The statement, $P(p - value_y \leq \epsilon)$, expresses the probability P that the *p-value*, derived from a set of i.i.d. instances, falls below or equals the user-defined significance level ϵ. This probability is constrained by the property in Eq. 2, signifying that the likelihood of obtaining a *p-value* less than or equal to ϵ is itself limited by ϵ. In practical terms, this encapsulates the assurance that, under the assumption of i.i.d. instances, the probability of observing a *p-value* leading to the rejection of the null hypothesis does not exceed the chosen significance level. Consequently, we may output a set of possible predictions and construct the prediction region C^ϵ, as follows:

$$C^\epsilon = \{y \in Y | p - value_y \geq \epsilon\} \tag{3}$$

Because of the property in Eq. 2, the probability that each set of predictions does not contain the correct class will be less than or equal to ϵ, so we limit the error rate to less than or equal to ϵ. In a binary classification problem with a positive and a negative class there are four possible outcomes for a conformal prediction i.e., positive, negative, both classes (positive and negative), and no class assignment (empty class). In each case, the classes are included in the prediction region (Eq. 3), when we are confident with the desired level. The "empty" label indicates that the sample lies outside the range where the model can make reliable predictions. In other words, the model cannot assign any class with the user-defined required confidence level, signifying that the sample is beyond the boundaries of the model's applicability. Consequently, the classification decision needs to be determined, by other in

silico methods and subsequently integrated into an enriched model. This step is useful for expanding the model's applicability domain (Alvarsson et al., 2021).

In a regression analysis framework, CP transforms point predictions from a model $\hat{f}$ trained on D data with n instances, to intervals which contain the true value with a level of guarantee defined by the user. In this case, to compute the *non-conformity* scores for every sample in the training set, we measure how different the observed y_i is from the model prediction $\hat{f}(x_i)$. A simple measure to calculate non-conformity is the absolute residual: $\alpha_i = |y_i - \hat{f}(x_i)|$. Given ϵ the user-defined significance level, we calculate the $Q_{1-\epsilon}$ quantile of the scores as:

$$Q_{1-\epsilon} = \frac{(1-\epsilon)(n+1)}{n}$$

For a new input x_{n+1}, the prediction interval is defined as follows:

$$[L(x_{n+1}), U(x_{n+1})] = [\hat{f}(x_{n+1}) - Q_{1-\epsilon}, \hat{f}(x_{n+1}) + Q_{1-\epsilon}]$$

where, L is the lower limit and U the upper limit for the new input x_{n+1}. The resulted prediction interval $[L(x_{n+1}), U(x_{n+1})]$, assuming data D are exchangeable, satisfies the property of marginal coverage:

$$P(y_{n+1} \in [L(x_{n+1}), U(x_{n+1})]) \geq 1 - \epsilon$$

In other words, the probability that the predicted value is included in the prediction interval is bigger or equal to the user-defined level of confidence.

1.1 Transductive conformal prediction

CP was originally used in the transductive or full version (Gammerman & Vovk, 2007; Vovk et al., 2005). The transductive CP (TCP) uses all the available data to train the model and thus, we can produce more accurate and informative predictions. After choosing the appropriate *non-conformity* function, we add the features of a new instance x_{n+1}, and assuming its class y_{n+1}, we retrain the model K times, where K is the number of all the possible classes for x_{n+1}. In a binary classification problem, the model will be trained 2*Z times for each class, with Z being the number of points in the test set. Then, for these two new training sets, we apply the *non-conformity* measure, we compute the *p-values* (Eq. 1) and finally, we check whether the features (x_{n+1}) of an instance in the test set "conforms" to the predictions of the training set and leads to decisions for the creation of the prediction region. TCP is a suitable method for analyzing small data sets as it works as an online framework. For larger datasets more computationally efficient methods should be selected e.g., inductive CP.

1.2 Inductive conformal prediction

Inductive CP (ICP) is the most popular CP approach. TCP has high a computational cost and may not be suitable for certain applications in genomic medicine. For example, multi-omics analyses usually involve large datasets due to the sheer size, complexity, and variability of the genomic data and the technologies that are used to produce it. To deal with this issue ICP trains the basic algorithm only once (Papadopoulos, 2008) by splitting the training set n into two smaller sets, a training set with $m < n$ and a *calibration set* with $n - m$ instances. The training set is used to create the "prediction region" and the instances in the *calibration set* are exclusively used to calculate the *p-value* of each possible class of a new test instance X. No matter which CP method we use, ICP will result in unbiased predictions. The efficiency of an ICP model depends on many factors such as, how large and well-constructed (i.i.d.) the dataset is, how effective is the underlying ML algorithm, and which *non-conformity* measure is employed.

References

Alvarsson, J., McShane, S. A., Norinder, U., & Spjuth, O. (2021). Predicting with confidence: Using conformal prediction in drug discovery. *Journal of Pharmaceutical Sciences*, *110*(1), 42–49. https://doi.org/10.1016/j.xphs.2020.09.055

Gammerman, A., & Vovk, V. (2007). Hedging predictions in machine learning. *The Computer Journal*, *50*(2), 151–163. https://doi.org/10.1093/comjnl/bxl065

Papadopoulos, H. (2008). Inductive conformal prediction: Theory and application to neural networks. In *Tools in artificial intelligence*. Citeseer. https://doi.org/10.5772/6078

Shafer, G., & Vovk, V. (2008). Algorithmic learning in a random world. *Journal of Machine Learning Research*, *9*(3).

Vovk, V., Gammerman, A., & Shafer, G. (2005). *Algorithmic learning in a random world* (Vol. 29). Springer.
